# Assessing Temperature-Induced Changes in Arthropod Communities over One Year: A Comparative Analysis

**DOI:** 10.3390/insects17030265

**Published:** 2026-03-01

**Authors:** Sophie Wallon, Gabor Pozsgai, Paulo A. V. Borges, Rui B. Elias

**Affiliations:** 1 CE3C—Centre for Ecology, Evolution and Environmental Changes/Azorean Biodiversity Group, CHANGE—Global Change and Sustainability Institute, School of Agricultural and Environmental Sciences, University of the Azores, Rua Capitão João d’Ávila, Pico da Urze, 9700-042 Angra do Heroísmo, Azores, Portugal; pozsgaig@coleoptera.hu (G.P.);; 2 Center of Applied Economic Studies of the Atlantic, University of the Azores, 9700-042 Angra do Heroismo, Azores, Portugal; 3 IUCN SSC Atlantic Islands Invertebrate Specialist Group, 9700-042 Angra do Heroísmo, Azores, Portugal; 4 IUCN SSC Monitoring Specialist Group, 9700-042 Angra do Heroísmo, Azores, Portugal

**Keywords:** ground-dwelling fauna, temporal trends, experimental warming, open top chambers, intensive grassland

## Abstract

Understanding how rising temperatures driven by climate change affect biodiversity and ecosystem functioning is particularly relevant in fragile island environments. Terceira Island, part of the Azorean Archipelago (Portugal) in the mid-Atlantic Ocean, was selected for an in situ experiment to assess the effects of simulated warming on arthropod diversity and grass productivity in intensively managed pastures. Over one year, we examined the influence of a simulated increase in temperature via Open Top Chambers (OTCs) on ground-dwelling arthropod assemblages and plant biomass. We used several statistical methods to analyze both individual variables and overall community patterns to explore the relationships between treatments, grass biomass, and arthropod community structure. Although the OTC treatment did not result in any significant changes to the analyzed variables, an increase in diversity indices was observed over the course of the experiment. Distance-based redundancy analysis revealed significant effects of both OTC treatment and altitude on arthropod community composition. Models showed that the analyzed variables only partially explained the observed changes. Nevertheless, additional factors, such as vegetation structure and microclimatic variation, may also contribute to the observed patterns and warrant further investigation. These results underscore the importance of long-term, multifactorial approaches to understanding interactions between warming, plant productivity, and arthropod diversity.

## Simple Summary

This study examined the effects of simulated warming on arthropod diversity and grass productivity in intensively managed pastures on Terceira Island, Azores. Simulating an increase in temperature via Open Top Chambers (OTCs), we found that warming did not significantly alter grass biomass or arthropod diversity, although diversity slightly increased over the year. Community composition was influenced by both treatment and altitude, but other factors like vegetation structure or microclimate may also play a role and need to be investigated. These findings highlight the need for long-term, multifactorial studies to understand warming effects on island ecosystems.

## 1. Introduction

Climate change is one of the most urgent global challenges, profoundly affecting ecosystems, biodiversity, and agricultural productivity worldwide [1,2,3]. Rising temperatures, shifting precipitation patterns, and more frequent extreme weather events are transforming both natural and managed ecosystems [4].

Volcanic islands also face intensified threats from sea-level rise, stronger storms, and altered ocean currents, which amplify the direct effects of climate change on terrestrial systems [5]. These impacts are especially severe in oceanic archipelagos such as the Azores (Portugal) [6,7,8], where geographical isolation, limited land area, and high endemism make ecosystems particularly sensitive to environmental change [5,9,10,11,12,13]. Unique habitats like the Laurisilva forests, shaped by specific climatic conditions, are highly vulnerable to even small variations in temperature and precipitation [5,9,10,11,14].

Such shifts endanger not only native species but also the ecological processes sustaining these fragile environments. For example, changes in rainfall can alter air humidity and soil moisture—key factors for the health of hyperhumid island ecosystems [15]. Such shifts also endanger associated organisms living in the habitat, such as arthropods and ectothermic animals, which are particularly vulnerable to shifts in temperature and habitat structure [16,17,18,19,20,21,22,23].

In regions like the Azores, where biodiversity conservation and agricultural sustainability are closely linked, temperature and precipitation changes may therefore have far-reaching ecological and socio-economic consequences.

Currently, the Azores’ agricultural landscapes, including pastures, are of great importance as they are crucial for livestock production and therefore for the local economy [24,25,26]. Pastures serve as an essential resource for livestock, but they are also dynamic systems that respond to environmental changes [27,28]. Indeed, interactions between plant communities and their associated arthropod fauna are crucial for maintaining ecosystem functions [29]. Of particular interest in these ecosystems, therefore, are arthropod organisms that contribute significantly to essential ecosystem services such as nutrient cycling, soil formation, decomposition, and pest regulation [30,31,32,33]. As a key component of the food web, arthropods are highly sensitive to environmental changes, making them valuable bioindicators for assessing ecosystem health [34,35]. Also, herbivorous arthropods that rely on grasses for food and shelter are central components of grassland ecosystems, providing a critical food source for higher trophic levels [36].

It is therefore essential to understand the responses of arthropod communities to climate change in grasslands to build knowledge and advise stakeholders on future land management (risk assessment, grass selection, pasture elevation).

Thus, this study simulated an increased temperature in situ and assessed its impact on arthropod communities during the one-year in situ experiment on the island of Terceira (Azores, Portugal). By collecting data from the first and second years of the study, we aim to gain insight into immediate changes in arthropod community composition and shed light on rapid ecological responses to changing environmental conditions.

The aims of the study are:

(i) To assess whether changes in arthropod community structure and species composition occur after one year of elevated temperature.
(ii) To identify whether temperature or grass biomass, or the combination of these, plays a significant role in these potential changes.

## 2. Materials and Methods

### 2.1. Study Area

**Figure 1:**
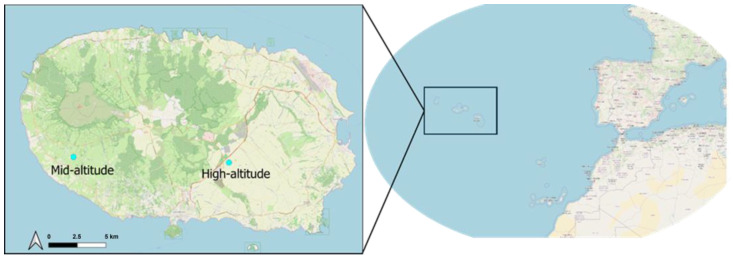
Location of the Azorean Archipelago, the island of Terceira, and the two experimental fields.

The study took place on two intensively managed pastures located on Terceira Island, part of the Azores archipelago in Portugal (between 38°37′ N and 38°48′ N in latitude and 27°02′ W and 27°23′ W in longitude) during the boreal summers of 2020 and 2021. The island’s area is 402 km^2^ and its maximum elevation is 1023 m. The two spatially independent study sites were located at 301 m above sea level (38.7016° N, −27.3258° W) and at 386 m above sea level (38.6978° N, −27.1701° W), hereafter referred to as the “mid-altitude” and “high-altitude” sites, respectively (Figure 1). Although the “high-altitude” site is far below the island’s peak (1023 m a.s.l.), it represents the upper elevation range within the study area. The two locations are classified as intensive pastures, with the mid-altitude field primarily covered by Italian ryegrass *(Lolium multiflorum* Lam., Poaceae) and the high-elevation field dominated by common velvet grass (*Holcus lanatus* L., Poaceae).

### 2.2. Experimental Design

The field experiment was conducted using Open Top Chambers (OTCs). OTCs are practical and effective tools in ecological research for simulating temperature increases, providing a controlled environment that mimics realistic warming effects while minimizing interference with other environmental variables. Their accessibility and simplicity make them especially useful for long-term field experiments studying the ecological impacts of climate change [37]. OTCs are constructed with panels that act as wind barriers, minimizing heat loss through convection. Their open top design allows rain to enter and air to circulate freely, creating small air vortices or eddies [37].

In each field, twenty 1 × 1 m plots were arranged in a grid, spaced 1.5 m apart. Ten plots were randomly selected to serve as controls, while the remaining ten were enclosed by OTCs. The OTCs were designed to cover the 1 × 1 m plots and included an additional 25 cm border around each plot [38]. This buffer zone allowed easy access for maintenance and monitoring while preventing damage to the plots. It also facilitated the placement of pitfall traps at each outer corner of the plots, all within the OTC boundaries. Additionally, the OTCs were elevated approximately 5 cm above ground, allowing crawling arthropods to move freely throughout the sampling area. To track temperature differences, data loggers (Easy Log: EL-USB-2) were placed both inside the OTCs and in the control plots. Over the two years, the average temperature in the OTCs was 1.2 °C (SD = 0.3 °C) higher than in the control plots (Table A1 and Table A2).

### 2.3. Arthropod Sampling and Identification

This research focused on arthropod communities found in intensively managed pastures. Since OTCs can act as barriers for flying insects and introduce bias, the study targeted ground-dwelling arthropods. Pitfall traps, which are designed to capture epigeal organisms crawling on the soil surface, were used for sampling. Four traps were placed at the outer corners of each plot, totaling 80 traps per field. In OTC plots, the traps were set in the 25 cm margin surrounding each plot within the OTCs (see also the Section 2.2). The traps consisted of 330 mL plastic cups, ca. 12 cm deep and 8 cm in diameter, filled with a solution of 20% ethylene glycol (car coolant) and a few drops of soap to break the surface tension. To protect the traps from rain, they were covered with plastic dishes raised on small metal supports, which left the trap openings accessible to arthropods. All collected specimens were preserved in 96% ethanol.

This analysis only used catch data from one of the four traps in each plot. Arthropod sampling was conducted in the summers of 2020 and 2021, with traps left open for 14 days. An exception occurred in the mid-altitude field during the summer of 2020, when traps were only functioning for 13 days. In this case, arthropod abundance was extrapolated to 14 days based on data from other traps, though species richness was not extrapolated.

All sampled arthropods were sorted and, where possible, identified to the species level for the following groups: Arachnida (including Araneae, Opiliones, and Pseudoscorpiones), Diplopoda, Chilopoda, and Insecta (excluding Diptera and Hymenoptera, but including Formicidae), and Lepidoptera. To each specimen that could not be identified to the species level, a morphospecies code was assigned. Initial sorting and identification were performed by the first author (SW) and a team of students acting as parataxonomists (see Acknowledgements), followed by verification by a taxonomic expert (PAVB). Species names and colonization status were based on the most recent checklist of Azorean arthropods [39]. Data analysis focused on the activity density of adult individuals identified to the morphospecies level. While earlier studies on Azorean arthropods (e.g., [38]) included juvenile spiders, this study excluded them due to the dominance of Erigoninae linyphiid spiders, which made the identification challenging.

All specimens are preserved and stored in the Dalberto Teixeira Pombo (DTP) Collection at the University of the Azores on Terceira Island. Data from 2020 and 2021 are publicly accessible in [38], available at https://ipt.gbif.pt/ipt/resource?r=pasturclim_otc&v=1.6 (accessed on 24 February 2026).

### 2.4. Grass Sampling

The experimental plots were established for the entire duration of the experiment. In 2020 and 2021, the grasses in each plot were manually harvested at the end of each season and after sampling arthropods with pitfall traps. Grass cover in each plot was visually estimated, with grass typically covering 80–100% of the area. Occasional minor disturbances (e.g., small patches removed by burrowing animals) were accounted for by calculating a standardized grass-cover ratio when comparing plots. These small variations did not affect arthropod sampling. For this analysis, only the grass productivity from the summer of both years was used. For each plot, the freshly harvested grass was weighed green and then dried in a drying oven at 60 °C to a constant weight (i.e., when all the water in the grasses had evaporated). The analysis was carried out using the dry mass of the grass, which will be referred to as “grasses” or “grass biomass” henceforth. Grass biomass data are presented in Appendix A (Table A3 and Table A4).

### 2.5. Data Analysis

The analysis of arthropod abundance and its relationship with environmental factors was conducted in R (version 4.2.3). The arthropod activity-density data (i.e., number of adult individuals caught), encompassing two summer seasons (2020 and 2021) and two fields (mid- and high-altitude), was used for the analysis (e.g., previous section “Arthropod Sampling and Identification”).

We analyzed the diversity and community composition of arthropods using a series of statistical models and visualizations (Table 1). The analysis aimed to explore the relationships between arthropod diversity, environmental factors, and treatment effects across two years.

Grass biomass was first analyzed to assess the effects of experimental warming and interannual variation and to justify its inclusion as a covariate in subsequent diversity models. Biomass was modeled as a continuous response variable using a linear mixed-effects model (LMM) fitted with the lmer() function from the lme4 package [40]. The treatment (control vs. OTC) and the year were included as fixed effects, while the field (representing elevation differences among sites) was included as a random effect. To quantify the diversity of arthropod assemblages, we employed the iNEXT R package, which estimates the first three Hill numbers (q = 0, 1, and 2) for multiple diversity indices. Hill numbers [41,42] are metrics used to quantify biodiversity by summarizing both the number of species and their relative abundances within a community into a single value. The importance assigned to dominant versus less dominant species varies with the value of the q parameter. Specifically, Hill number 0 corresponds to species richness, whilst Hill numbers 1 and 2 correspond to the exponential of the Shannon entropy and the inverse of the Simpson index, respectively. Hill number 1 captures the effective number of species by weighing the contribution of each species according to its abundance, while Hill number 2 emphasizes the dominance of the most abundant species, highlighting the influence of species richness, evenness, and dominance on community structure. This approach allows for a comprehensive assessment of species richness, Shannon diversity, and Simpson diversity across treatment groups and years. Hill numbers were computed using bootstrapping techniques (*n* = 500) to ensure robust estimates.

Shannon (q = 1) and Simpson (q = 2) diversity indices were analyzed using linear mixed-effects models (LMMs) with Gaussian error distributions. Treatment (control vs. OTC), year, and grass biomass were included as fixed effects. The field was included as a random effect to account for differences among sampling fields. Because elevation was confounded with field identity (i.e., one field per elevation), elevation was not tested as a fixed effect but was instead accounted for through the random effect of field. Model assumptions were assessed using residual diagnostics, and model fit was evaluated using marginal and conditional coefficients of determination (R^2^).

Species richness (q = 0), expressed as count data, was initially analyzed using a generalized linear mixed-effects model (GLMM) with a Poisson error distribution. However, inclusion of the field as a random effect resulted in singular model fits due to the limited number of field levels. Consequently, species richness was analyzed using generalized linear models (GLMs) with Poisson error distribution and without random effects. Two separate models were fitted to assess (i) interannual variation and (ii) experimental warming effects (Table 1).

To evaluate the influence of the treatment on arthropod community composition, we performed a distance-based redundancy analysis (dbRDA) using the capscale() function from the vegan package [42]. Prior to the analysis, arthropod abundance data were Hellinger-transformed with the decostand() function to reduce the weight of rare taxa. The dbRDA model was fitted with treatment and (experimental) field (including their interaction) as explanatory variables, while sampling year was included as a conditional variable to control for temporal heterogeneity. Euclidean distance was used as a dissimilarity measure. The explanatory power of the model was evaluated using the R^2^ and adjusted R^2^ statistics. The significance of the treatment effect was assessed through a permutation-based ANOVA with 999 permutations.

To further explore patterns within each sampling location, two additional dbRDA models were fitted separately for mid-altitude and high-altitude fields, using the treatment as the explanatory variable and the sampling year as a covariate. This allowed visualization of treatment effects within each field independently. The three species contributing most to treatment differences were identified using a SIMPER analysis [43] and were visualized as vectors in ordination plots. Ordination plots were created using the ggplot2 package [44] for visualization.

## 3. Results

A total of 7951 adult arthropod specimens were collected from two fields over two summers (2020 and 2021), belonging to 4 classes, 17 orders, 49 families, and 106 morphospecies. A total of 89 of these morphospecies were identified to the biological species level (which will be referred to as “species” in this paper), while 17 were identified at the order, family, or genus level. The 88 species-level identifications represent about 97% of the specimens (n = 7780).

### 3.1. OTC Influence on Grass Biomass

**Figure 2:**
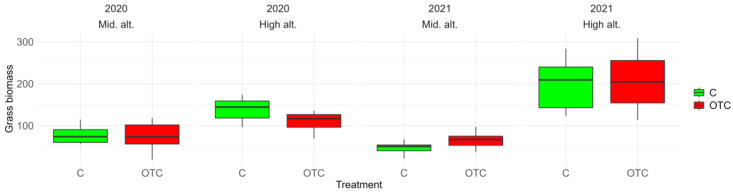
Boxplots of grass biomass (g) by sampling year, field, and treatment. “C” and “OTC” correspond respectively to control and Open Top Chamber.

The estimated mean biomass for the controls was approximately 115.96 g (SE ± 10.80), while the mean for the OTC treatment was around 114.44 g (SE ± 15.27) (Figure 2). Grass biomass was first analyzed as a response variable to justify its inclusion as a covariate in the diversity models. The model indicated that the year had a significant effect on grass biomass, with an estimated increase of 28.78 g (SE = 10.15; t = 2.84; *p* < 0.01), whereas the treatment had no significant effect (estimate = −1.52 g; SE = 10.15; t = −0.15; *p* = 0.88).

### 3.2. Effect of Year, Treatment, and Biomass

**Figure 3:**
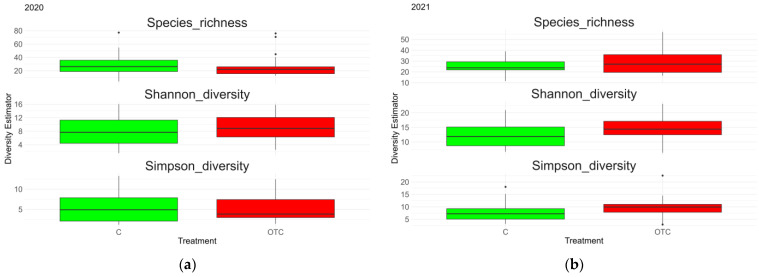

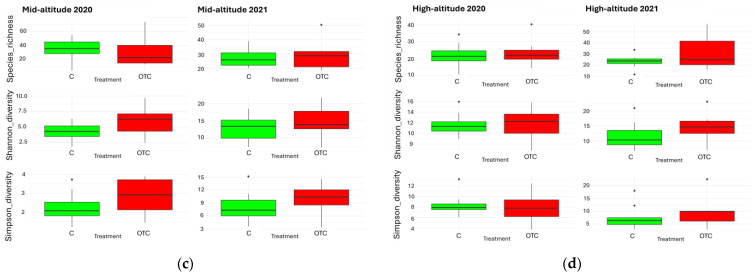
Boxplots showing the different diversity indices, species richness (q0), exponential Shannon index (q1), and the inverse Simpson index (q2) in (**a**) 2020 mid and high altitude combined, (**b**) 2021 mid and high altitude combined, (**c**) mid altitude 2020 and 2021 and (**d**) high altitude 2020 and 2021. “C” and “OTC” correspond respectively to the control plot and Open Top Chamber.

The results of the different models are presented in Table 2. The year of sampling was a significant predictor for species richness, Shannon diversity, and Simpson diversity. However, the treatment and the grass biomass showed no significance with the diversity indices analyzed. The model explained 22.1% of the variance in Shannon diversity based on fixed effects alone (marginal R^2^ = 0.221) and 50.9% when including the random effect of field (conditional R^2^ = 0.509). For the Simpson diversity, fixed effects (year and grass biomass) explained 17.8% of the variance in Simpson diversity (marginal R^2^ = 0.178), while the inclusion of the field as a random factor increased the explained variance to 46.3% (conditional R^2^ = 0.463), indicating a substantial variation as well among fields. These patterns are also illustrated in Figure 3.

The model to test the interannual variation in species richness showed that the year of sampling was a significant predictor. However, the model explained only a small proportion of the variance (McFadden’s pseudo-R^2^ = 0.012), indicating that while sampling year had a detectable effect, its overall influence on species richness was limited. Grass biomass did not have a significant effect, suggesting that species richness remained relatively stable between years, even though subtle changes in community composition or evenness may have occurred.

The model testing the effect of experimental warming on species richness showed extremely low explanatory power (McFadden’s pseudo-R^2^ = 0.00006), indicating that treatment and grass biomass explained virtually none of the variation in species richness. Neither treatment (*p* = 0.999) nor plant material (*p* = 0.868) had a significant effect on species richness. These results suggest that species richness was not influenced by experimental warming and remained relatively stable across treatments.

### 3.3. Distance-Based Redundancy Analysis

#### 3.3.1. Middle and High Altitude Combined

**Figure 4:**
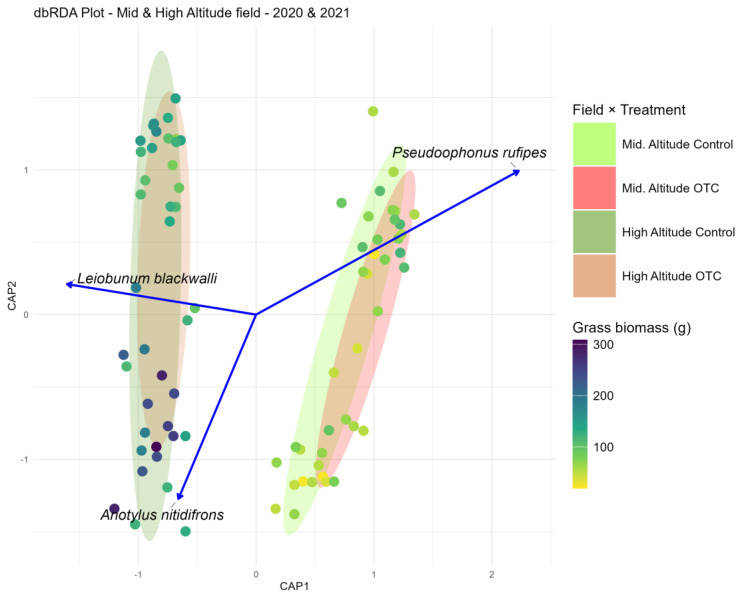
Ordination plot to represent the arthropod community while considering both fields and years together. Grass biomass for each sampling field and year was plotted following a color gradient. Vectors represent species that most influenced the community: *Anotylus nitidifrons*; *Pseudoophonus rufipes; Leiobunum blackwalli*.

The dbRDA including summer 2020 and 2021 data from both the mid- and high-altitude fields (Figure 4) explained 47.7% of the variation in arthropod community composition (constrained inertia = 19.67, total inertia = 41.27; R^2^adj = 0.477). The remaining 51.7% of the variation was left unexplained by the model. The first two constrained axes accounted for 88.7% of the explained variance (CAP1 = 58.4%, CAP2 = 30.3%). CAP1 mainly represented differences between the two fields (altitudinal levels), while CAP2 captured additional variation related to the treatment (control vs. OTC) within fields. Although the previous analysis (see above) showed no response of grass biomass to the treatment during the summers of 2020 and 2021, the plotting of the grass biomass on the ordination plot highlighted a higher grass biomass on the field at higher elevation, which was expected.

Permutation ANOVA confirmed that the overall model was highly significant (F = 23.067, *p* < 0.001). Sequential tests showed significant effects of treatment (F = 8.441, *p* < 0.001), experimental field (F = 21.602, *p* < 0.001), and their interaction (F = 39.159, *p* < 0.001). Several taxa (e.g., *Anotylus nitidifrons* (Wollaston, 1871)—Coleoptera; *Pseudoophonus rufipes* (De Geer, 1774)—Coleoptera; *Leiobunum blackwalli* Meade, 1861—Opiliones) were strongly associated with these axes, highlighting their contribution to observed patterns. Overall, both the field and interaction between warming treatment and field jointly structured ground-dwelling arthropod assemblages.

#### 3.3.2. Middle-Altitude Field

**Figure 5:**
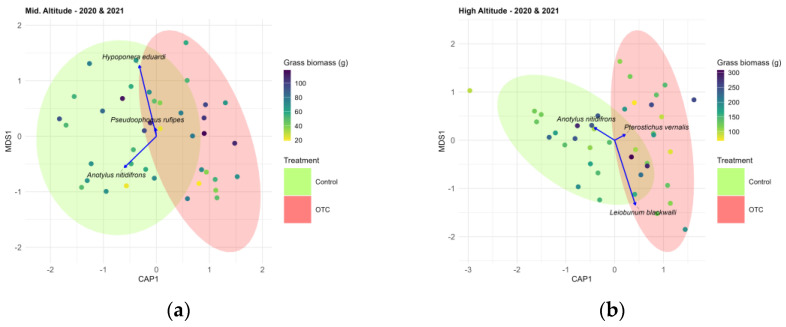
(**a**) Ordination plot to represent the arthropod community while considering the mid-altitude field and both years. Grass biomass for each sampling site and year was plotted following a color gradient. Vectors represent species that most influenced the community: *Anotylus nitidifrons* (Wollaston, 1871)—Coleoptera; *Pseudoophonus rufipes* (De Geer, 1774)—Coleoptera; *Hypoponera eduardi* (Forel, 1894)—Hymenoptera. (**b**) Ordination plot to represent the arthropod community while considering the high-altitude field and both years. Grass biomass for each sampling site and year was plotted following a color gradient. Vectors represent species that most influenced the community: *Anotylus nitidifrons* (Wollaston, 1871)—Coleoptera; *Pterostichus vernalis* (Panzer, 1796)—Coleoptera; *Leiobunum blackwalli* Meade, 1861—Opiliones.

The dbRDA model that was conducted for the mid-altitude field (Figure 5a) explained 5.7% of the total variance in the assemblage data, with an adjusted R^2^ of 4.1%. The ordination revealed a clear separation between the control and OTC treatment along the first canonical axis (CAP1), which accounted for all the constrained variation. The effect of the treatment was statistically significant (F = 3.35, *p* < 0.001), indicating that warming induced by the OTCs influenced the composition of ground-dwelling arthropod communities in this field. For instance, rove beetle (Coleoptera: Staphylinidae), *Anotylus nitidifrons*, showed higher associations with control plots. However, as the model only explained 5.7% of the total variance in the assemblage, the observed pattern was influenced by other variables to a great extent.

#### 3.3.3. High-Altitude Field

The dbRDA model that was conducted for the high-altitude field (Figure 5b) explained 7.1% of the total variation in community composition, with an adjusted R^2^ of 0.054. The effect of the treatment was statistically significant (F = 3.92, *p <* 0.001). Species such as the beetle *Pterostichus vernalis* were positively associated with OTC treatment, whereas the Staphylinidae *Anotylus nitidifrons* showed higher associations with the control. However, as with the mid-altitude field, the model only explained a small proportion of the observed pattern, suggesting that other variables may play a more significant role in shaping the arthropod community.

## 4. Discussion

The present study aimed to assess the effects of OTCs on grass biomass and arthropod diversity between two different years (2020 and 2021). Our initial findings regarding summer grass productivity indicated that the treatment did not significantly affect grass biomass during this season. These results are aligned with previous studies, which have shown that the responses of plant communities to simulated climate change can vary widely based on specific environmental factors, such as rain patterns [45], which can easily mask treatment effects over a relatively short period of time. As our experiment did not include rain shelters, we can assume that other environmental factors during the summer, for instance the amount of rainfall, may affect the structure of the grass more than the increased temperature within the OTCs. Also, in contrast to more mobile organisms such as arthropods, plant communities often exhibit slower responses to changes in their environment [46,47], which may explain the lack of significant response in the relatively short time frame of our experiment. However, in our experimental fields, we did observe higher grass biomass inside the OTCs at mid altitude when considering the grass biomass during the entire years of 2020 and 2021 and not only summer productivity [48] (p. 19). Roth et al. (2023) [49] also demonstrated that even modest temperature increases of +0.5 °C can alter competitive dynamics in grasslands, favoring productive grass species. In their study, however, the overall biomass response was limited, likely due to the mild warming effect and the experiment’s short duration.

Our results also indicate that temporal variation (year) had a measurable effect on arthropod diversity. Both Shannon diversity and Simpson diversity were significantly influenced by the year of sampling, and species richness showed a detectable response as well, although the explanatory power of the models for richness was very low. This suggests that while interannual environmental fluctuations may influence community composition and evenness, total species richness remains relatively stable over time.

In contrast, experimental warming via OTCs did not have a significant effect on species richness, nor did grass biomass, with the models explaining virtually none of the variation. This indicates that ground-dwelling arthropod communities in these pastures are relatively resilient to short-term warming and local differences in grass biomass. Notably, the inclusion of the field as a random factor in the Shannon and Simpson models increased the explained variance substantially, highlighting that spatial heterogeneity among pastures and altitude remains a key driver of arthropod diversity. Overall, these results suggest that while community evenness and composition may shift slightly between years, species richness is largely stable, and short-term warming or variations in plant biomass alone are insufficient to drive major changes in these arthropod assemblages.

This contrasts with some previous studies suggesting that increased plant biomass can provide additional resources and enhance habitat complexity, thereby supporting greater arthropod diversity [50,51,52,53]. Such relationships are often explained by the provision of more food resources for herbivorous arthropods and enhanced refuge from predation, which can in turn support higher predator biomass and diversity through bottom-up trophic cascades [54,55]. Increased structural complexity from greater vegetation can also create a wider array of microhabitats, allowing for niche partitioning among arthropod species [56]. While these mechanisms are well-supported in the literature, our findings suggest that, in these pasture systems, variations in grass biomass alone are insufficient to drive measurable changes in arthropod species richness or diversity.

However, the observed interannual variability in diversity indices underscores the potential for these communities to respond to longer-term environmental shifts, particularly as ectothermic organisms are known to exhibit variable physiological and metabolic responses to heat stress depending on their specific traits and developmental stages [57]. Consequently, understanding how these physiological constraints interact with changing precipitation patterns is essential, as rainfall regimes have been demonstrated to significantly influence species composition and multi-trophic interactions within arthropod communities [58]. Specifically, research indicates that arthropod community composition is largely determined by seasonal rainfall and plant cover, with detritivore groups being particularly sensitive to precipitation patterns [58]. This sensitivity is further corroborated by studies showing that precipitation and temperature act as key climatic drivers regulating species diversity and plant coverage through complex direct and indirect pathways [59]. These findings emphasize that the impacts of climate change on ground-dwelling arthropods are likely mediated not just by thermal increases, but by the interplay of multiple abiotic factors that structure habitat availability and resource quality over time [59,60].

When looking at arthropod diversity, the observed pattern with an increase in the diversity indices from the first to the second sampling year should be interpreted with caution as the difference may reflect natural interannual variability rather than a genuine temporal trend, given the short duration of the experiment. Many pasture-dwelling arthropods in the Azores are habitat generalists that can rapidly respond to environmental fluctuations and management intensity. Long-term monitoring in agricultural systems elsewhere has shown that arthropod richness can fluctuate considerably between years, with only marginal overall increases over time [61]. But the increased temperature inside the OTCs did not significantly alter diversity metrics. These results are consistent with those by Wallon et al. (2023) [60], who found no significant changes in arthropod diversity between treatments (OTCs versus control) when considering all species collected across various seasons within the same year. This lack of treatment effects challenges the assumption that increasing temperature in the magnitude that can be achieved via OTCs would lead to substantial changes in arthropod diversity. However, Hu et al. (2025) [62] reported that even modest experimental warming can significantly reduce arthropod species diversity and biomass. They highlighted that the effects of temperature may depend strongly on local environmental conditions and vegetation responses. Such findings also support the idea that other ecological factors, such as fluctuating humidity, rainfall patterns, or vegetation, may play a more important role in shaping community dynamics than the direct effects of increased temperature [63,64]. A longer-term experiment might also detect slower changes in the ecosystem that our experiment could not because of its short duration. Extending the duration of the experiment would therefore be essential to determine whether the observed variation represents a consistent response to warming or merely short-term variability.

On the other hand, the dbRDA results indicate that both experimental field and OTC warming influenced the composition of ground-dwelling arthropod assemblages, though the magnitude of these effects varied between fields. When both altitudinal levels were analyzed together, the model explained nearly half of the total variation in community structure, demonstrating that species composition was strongly field-dependent. The strong statistical support for the interaction between the treatment and the fields suggests that warming effects may have been modulated by altitude, with species responses differing between mid- and high-elevation environments. The altitudinal modulation of warming effects also highlights the complexity of ecological responses to climate change, particularly as elevational gradients often drive significant variation in microclimates and vegetation, influencing arthropod distributions [65]. Such variations in microclimates along altitudinal gradients can create distinct community structures, highlighting the influence of temperature and other climatic variables on species distribution [65,66]. This suggests that arthropod communities at different elevations may exhibit distinct sensitivities to thermal shifts, potentially leading to differential community restructuring under future warming scenarios [67].

Among the species identified as indicators, *Anotylus nitidifrons* stood out as particularly interesting due to its ecological characteristics. This common island species, known for its high dispersal capacities and attraction to light [39,68,69], has been observed in various types of decaying vegetation and, less frequently, in dung [69]. In our study, *A. nitidifrons* tended to be associated with the control plots across both altitudes. Although little is known about the biology of this species or even its genus, a few possible explanations can be proposed. Its attraction to light could have naturally drawn it to the control plots, which tended to be slightly more luminous than the OTCs. Alternatively, a higher amount of decaying vegetation in the control plots could have provided more suitable conditions for its occurrence. In contrast, *Pterostichus vernalis*, a common and abundant carabid predator in agricultural areas in Europe [70,71] and considered introduced in the Azores [39], was more frequently associated with OTC plots at higher elevations. This pattern may be linked to the species’ sensitivity to microclimatic conditions, as *P. vernalis* is known to thrive in environments with higher temperatures and humidity [72], which can enhance its metabolic efficiency and reproductive success [70]. These traits may explain its higher abundance in OTCs, where such conditions are likely to be more prevalent. Different responses across species and sampling sites highlight the intricate interplay between localized warming effects and broader environmental factors in shaping community dynamics [73]. Overall, the dbRDA analyses highlighted that experimental warming exerts a measurable influence on arthropod communities, but this influence is highly field-dependent, most likely depending on the local species pool and the surrounding landscape matrix.

The modest explanatory power of some models highlights the ecological complexity of these systems and suggests that additional environmental variables, such as soil moisture, vegetation cover, prey availability, and biotic interactions, should be integrated into future models to better understand the mechanisms driving the observed patterns. Factors like inter- and intraspecific competition, trophic interactions, food resource availability, and seasonality also warrant further investigation [74].

While this study contributes to understanding the effects of climate change on grass biomass and arthropod diversity, it is important to acknowledge certain limitations. The two-year study period could not capture long-term trends, and the results are likely influenced by site-specific factors.

Long-term monitoring is essential to distinguish between short-term plastic responses and potential evolutionary adaptation, as arthropod populations face compounding pressures from land use changes and climatic warming that have far-reaching implications for ecosystem functioning [75]. Because such studies are still uncommon in the Azores, especially in intensive pastures, long-term research is urgently needed to examine how climate change affects diverse organisms by relating their seasonal population dynamics to key environmental variables, thereby revealing the mechanisms behind these trends.

## Figures and Tables

**Table 1:** Statistical models used for plant biomass and arthropod diversity analyses. Linear mixed-effects models (LMMs) were applied to continuous response variables, while generalized linear models (GLMs) were used for species richness. Field (elevation) was included as a random intercept when supported by the data.

Variable	What Was Tested ?	Which Model Was Used ?	Why ?
Plant Biomass	Effect of Treatment and Year	LMM (Gaussian) Fixed: Treatment + Year Random: Field (Elevation)	To test how experimental warming or interannual variation affects grass biomass while accounting for differences between fields
Species richness (Hill q = 0)	Interannual variation in diversity	GLM (Poisson) Fixed: Year + Grass biomass	Mixed-effects models including field resulted in singular fits; a GLM was used to robustly assess temporal effects
	Effect of experimental warming (OTC)	GLM (Poisson) Fixed: Treatment + Grass biomass	Random effects could not be reliably estimated for count data with limited grouping levels
Shannon diversity (Hill q = 1)	Combined effects on diversity	LMM (Gaussian) Fixed: Treatment + Year + Grass biomass Random: Field (Elevation)	Model was not singular and allowed accounting for elevation-related grouping
Simpson diversity (Hill q = 2)	Combined effects on diversity	LMM (Gaussian) Fixed: Treatment + Year + Grass biomass Random: Field (Elevation)	Model was not singular and allowed accounting for elevation-related grouping

**Table 2:** Results from linear and mixed models assessing the effects of the year on species diversity levels, including species richness, Shannon diversity, and Simpson diversity.

Diversity Index	Model	Effect	Estimate	Std. Error	t Value	*p*-Value
Shannon	LMM	Treatment	0.888	0.652	1.362	0.177
		Year	3.827	0.684	5.596	<0.001
		Grass biomass	−0.010	0.007	−1.403	0.165
Simpson	LMM	Treatment	0.672	0.594	1.131	0.262
		Year	2.954	0.623	4.741	<0.001
		Grass biomass	−0.010	0.007	−1.462	0.148
Species richness	GLM (interannual variation)	Year	0.126	0.055	2.285	<0.05
		Grass biomass	0.000	0.000	−0.319	0.750
Species richness	GLM (experimental warming effects)	Treatment	0.000	0.054	0.002	0.999
		Grass biomass	0.000	0.000	0.166	0.868

## Data Availability

Data from summer 2020 & 2021 used for this publication are available at Wallon et al. (2025)) [76]. Version 1.5. Universidade dos Açores. Sampling event dataset https://doi.org/10.15468/u2xh5g accessed via GBIF.org on 19 July 2024. Data from summer 2021 are available on request.

## Abbreviations

The following abbreviations are used in this manuscript:

OTC	Open Top Chamber
dbRDA	distance-based redundancy analysis

## Appendix A

**Table A1:** Average temperature (ºC) in control and Open Top Chambers (OTCs) for mid- and high-altitude fields during the summers of 2020 and 2021.

**Plots**	**Summer**	**Mid. Alt.**	**High. Alt.**
Control	2020	22.25	20.54
OTC	2020	23.88	21.43
Control	2021	22.02	20.64
OTC	2021	23.75	21.32

**Table A2:** Average temperature (ºC) differences between control plots and Open Top Chambers (OTCs) for mid- and high-altitude fields during the summers of 2020 and 2021. Mean temperature differences between altitudes and the overall mean difference across fields and years are also presented.

Summer	Mid. Alt.	High. Alt.	Mean Difference Between Fields (Altitude)
2020	1.63	0.9	1.26
2021	1.73	0.68	1.21
Average dif. 2020 & 2021	-	-	1.23

**Table A3:** Dry grass mass (g) for the 10 control plots (C) and 10 Open Top Chambers (OTCs) at mid altitude for the summers of 2020 and 2021.

Mid-Alt.	C 1	C 2	C 3	C 4	C 5	C 6	C 7	C 8	C 9	C 10
2020	83.7	68.4	56.34	71.2	92.91	107.1	57.36	76.3	114.3	57.24
2021	67.5	22.6	53.1	66.9	49.69	51.6	21.52	54.4	47.7	37.8
**Mid-Alt.**	**OTC 1**	**OTC 2**	**OTC 3**	**OTC 4**	**OTC 5**	**OTC 6**	**OTC 7**	**OTC 8**	**OTC 9**	**OTC 10**
2020	53.9	96.21	70.83	19.2	75.98	38	103.7	118.56	107.8	63.36
2021	67.83	68.58	77.13	37.43	57.38	47.9	78.6	66.24	97.3	51.9

**Table A4:** Dry grass mass (g) for the 10 control plots (C) and 10 Open Top Chambers (OTCs) at high altitude for the summers of 2020 and 2021.

High-Alt.	C 1	C 2	C 3	C 4	C 5	C 6	C 7	C 8	C 9	C 10
2020	140.75	143.44	102.89	145.57	96.35	153.33	174.11	160.86	170.05	110.82
2021	122.61	239.79	129.85	122.67	181.9	227.39	243.07	240.26	283.66	191.3
**High-Alt.**	**OTC 1**	**OTC 2**	**OTC 3**	**OTC 4**	**OTC 5**	**OTC 6**	**OTC 7**	**OTC 8**	**OTC 9**	**OTC 10**
2020	127.88	132.51	93.6	111.56	104.41	122.65	69.56	121.7	82.45	135.53
2021	308.7	255.63	122.55	255.02	221.37	285.37	113.9	182.54	144.99	187.86
